# Identifying and detecting facial expressions of emotion in peripheral vision

**DOI:** 10.1371/journal.pone.0197160

**Published:** 2018-05-30

**Authors:** Fraser W. Smith, Stephanie Rossit

**Affiliations:** School of Psychology, University of East Anglia, Norwich, United Kingdom; University of Toyama, JAPAN

## Abstract

Facial expressions of emotion are signals of high biological value. Whilst recognition of facial expressions has been much studied in central vision, the ability to perceive these signals in peripheral vision has only seen limited research to date, despite the potential adaptive advantages of such perception. In the present experiment, we investigate facial expression recognition and detection performance for each of the basic emotions (plus neutral) at up to 30 degrees of eccentricity. We demonstrate, as expected, a decrease in recognition and detection performance with increasing eccentricity, with happiness and surprised being the best recognized expressions in peripheral vision. In detection however, while happiness and surprised are still well detected, fear is also a well detected expression. We show that fear is a better detected than recognized expression. Our results demonstrate that task constraints shape the perception of expression in peripheral vision and provide novel evidence that detection and recognition rely on partially separate underlying mechanisms, with the latter more dependent on the higher spatial frequency content of the face stimulus.

## Introduction

Facial expressions of emotion are signals of high biological value. They are thought to have evolved in part to serve a critical communicatory function between conspecifics (e.g. [1–2]). Facial expressions transmit signals about the expresser’s emotion, intentions and environment and as such are proposed to play a key role in successful social interaction (see e.g. [3]). It has been argued that the evolution of the facial expression signaling system assisted adaptation ([4–5]). Hence the successful transmission and decoding of such signals by human agents is of much importance (see [6–9]).

While the basic facial expressions of emotion are universally recognized to a degree (i.e. happiness, sadness, fear, disgust, anger, sad, surprise and contempt; e.g. [10]) more recent studies have revealed that there are also cross-cultural differences in perception (e.g. [11–12]). Crucially most studies investigating expression perception have only tested performance under limited viewing conditions: most often, full-frontal face images from a relatively close viewing distance (see e.g. [13–14]). If, however, facial expressions are signals of high biological value then it is important to consider how well these signals can be recognized or detected across different viewing conditions (see [2, 3]), including how well they can be detected or recognized in peripheral vision. Although the face has been much studied in terms of relatively proximal communication, it has been argued that more attention needs to be paid to its role in signal transmission across a wider range of viewing conditions: e.g. from far distances[6], profile views [3], and we would add when first glanced via peripheral vision (see also [2–3, 15]).

Being able to recognize or detect facial expressions first glimpsed in peripheral vision may confer adaptive advantages such that an important social signal can be successfully resolved in a more efficient and rapid manner (see [3,16]). However, at the same time, reduced signal clarity (see also [3]) in peripheral vision will mean that perception is likely worse than when the signal is presented in central vision. In peripheral vision the visual system will be pushed to rely on the lower spatial frequency (LSF) content of the stimulus ([17–20]), due to the distribution of cone vs rod photoreceptors across the retina [21]. Thus expression recognition and detection performance should certainly be worse in peripheral vision than in central vision.

Of note, it has been proposed that a sub-cortical visual pathway might mediate rapid processing of faces and particularly those signalling facial expressions of fear, which conveys environmental threat (e.g. [22–25]). One of the core features of this pathway is thought to be its reliance on the LSF content of the stimulus (e.g. [23] but see also [26]). Evidence from blindsight [25] (see also [27]), tDCS [28] and human neuroimaging [24] have all implicated this pathway in the processing of fearful faces (but see [26] who found an effect non-specific for fear or to LSF content). While some work has shown stronger responses in the Amygdala, a target of the sub-cortical route, to LSF fearful (vs neutral) expressions ([24] and to fearful eyes in particular [29], it is important to note that other authors have argued that hypothesizing a sub-cortical route in humans is not necessary to explain existing data [30]. If, however, the sub-cortical route is present in humans, and offers adaptive advantages such as the faster detection of danger in the visual periphery, it is conceivable that fearful faces (signaling environmental threat) may be better recognized or detected at such a location, compared to other facial expressions (see also [16] for a similar argument and some supportive evidence).

While facial expression recognition has been much studied in central vision e.g.[6,8,32], much less attention has been given to studying recognition in peripheral vision. However as mentioned earlier, if facial expressions have high signal value, see e.g.[2–3], then it is important to investigate recognition across various viewing conditions, including when faces appear in peripheral vision. Previous research has shown that recognition of certain expressions is impaired in near peripheral vision while others are not [33–34]. Goren & Wilson [33], using synthetic faces scaled for cortical magnification, found that recognition of anger, sad and fear were all impaired in the periphery (an eccentricity of near 8 degrees) compared to foveal presentation. However this was not the case for happy faces. Calvo et al [34] used photographic faces (unscaled) and revealed that recognition was impaired for each basic expression category in the periphery (up to 6 degrees) except happy. Thus the only two previous studies that have examined recognition of expression in peripheral vision have used a rather limited range of eccentricities, in fact only extending into near peripheral (para-foveal) vision. One additional study has investigated how well facial expressions can be detected in peripheral vision (i.e. discriminated from neutral). In [16], the authors presented photographic faces (unscaled) at eccentricities up to 40 degrees, and revealed that emotion detection (fear Vs neutral & disgust Vs neutral) was present at 40 degrees in the periphery and was better than gender discrimination. Thus some information about expressive faces seems to be present relatively far into periphery (i.e. 40 deg), but whether that suffices for *expression recognition* as opposed to *expression detection* is an open question to date. Moreover, it is unknown which of the basic expression categories are best detected in peripheral vision (in [16], just tested two out of six). This is an important question for any account that proposes that particular expression signals (such as fear) may be preferentially processed in peripheral vision.

In the present study, for the first time, we investigate how recognition and detection of each basic expression category (happiness, sadness, fear, disgust, anger, sadness and surprise) changes as a function of eccentricity, up to 30 degrees in peripheral vision, in the same set of participants. Based on our previous work [6] and that of others [33–34], we expected happy and surprised faces to be well recognized and detected in peripheral vision. However, we also suspected that some expressions may be well *detected* in peripheral vision even though they may not be well recognized. This is because recognition and detection place different demands on the visual system and have been found to dissociate in multiple visual domains to date (e.g. object perception, face perception; see e.g. [35–36]). In particular the nature of the task that is performed with any particular visual stimulus set, such as the basic facial expressions, can *change* the visual information that is required to successfully perform the task, see[31, 37]. Hence in addition to investigating the signal value of the basic facial expressions when presented in peripheral vision, the present study investigates the extent to which two key processing stages of emotional face perception, i.e. detection and recognition, lead to dissociable patterns of performance, see also [38]. If both detection and recognition rely on a similar use of stimulus based SF information in faces then we would expect to observe similar effects of emotion as a function of eccentricity in both tasks.

Importantly in the present investigation we did not set out to test or control for the cortical magnification factor, see e.g. [20, 39]–which requires scaling images with increasing eccentricity to compensate for reduced cortical area devoted to processing. This was because we were primarily interested here in determining the signal value of the basic facial expressions of emotion under a comparable situation as to when they would first appear in our peripheral vision, before potentially being subsequently fixated: i.e., the size of the face does not change from when it first appears in our peripheral field until we potentially make a saccade to foveate the face. For the same reasons we did not control the SF content of our expressive faces, as whilst again this would be interesting from the point of view of underlying neural mechanisms, it would alter the potential signal value of particular expressions under particular viewing conditions.

## Method

### Participants

14 (7 male and 7 female) right-handed healthy volunteers between the ages of 19–26 (average age = 22.2, standard deviation = 1.89) years were recruited to take part. The study was approved by the Glasgow Caledonian University Ethics Committee for Life Sciences, and was conducted in accordance with the principles of the Declaration of Helsinki.

### Stimuli & design

The experiment used stimuli from the Pictures of Facial Affect [14] consisting of 5 male and 5 female faces in grayscale, showing expressions of happiness, sadness, fear, disgust, surprise, anger and neutral. The visual angle of the stimuli was 6.5 degrees (height). Stimuli were normalized for global luminance and contrast, by equalizing the pixel-wise mean and standard deviation across all images. A mask stimulus was also created by combining a random phase distribution with the average amplitude spectrum across all face stimuli, see [6, 40] through the use of Fourier Analysis. The stimuli were displayed on a Dell UltraSharp U2412M 61cm (24") LED monitor monitor driven by an OptiPlex 790 SF (graphics card 1GB AMD Radeon HD 6450) by means of the MATLAB Psychophysics Toolbox [41–42].

The order of the two experiments was counter-balanced across participants, with half of the participants carrying out the discrimination task first and the other half first carrying out the detection task. In the discrimination task faces were presented both centrally and to the left and right by 15 or 30 degrees. Each face identity displayed 7 different expressions (6 emotions and neutral), and each of these stimuli was presented once at each of the 5 eccentricities. This meant each identity appeared on the screen 35 times (7 expressions x 5 eccentricities). Owing to the fact that we used 10 different facial identities, and that each identity portrayed 7 different facial expressions, a total of 70 face presentations occurred at each eccentricity, making 350 trials in total. The participant was instructed to keep fixated on a fixation cross, and following the presentation of a face stimulus, decide which emotion (happy, sad, surprised, fearful, disgusted, angry or neutral) was displayed by pressing a corresponding keyboard key, as indicated on a response screen after each face presentation. There were two expression to response key mappings that were counter-balanced across participants (Mapping 1: Z = Surprise, X = Anger, C = Happy, V = Fear, B = Sad, N = Neutral, M = Disgust; Mapping 2: Z = Happy, X = Surprise, C = Fear, V = Sad, B = Neutral, N = Disgust, M = Anger). The participants were advised to answer as accurately as possible and were told to guess if unsure. The testing session was split into 6 blocks of trials, with the face stimuli presented in a random sequence, and lasted approximately 25 minutes.

The detection task was similar, with faces again being presented centrally or 15/30 degrees left or right. In this task however, participants were asked to state whether the face displayed an emotion or not. Every facial identity had 6 different emotions, which were presented once at every location. To keep trial numbers per condition (Emotional Vs Neutral) balanced, their neutral face was repeated 6 times at every location, meaning each identity was presented 12 times at each eccentricity. Thus at each eccentricity 120 facial stimuli were presented, and in total each identity was presented 60 times (6 expressive + 6 neutral x 5 eccentricities) throughout the task. Participants were advised to try and answer as quickly and as accurately as possible. Overall this task consisted of 600 face presentations (60 emotions+60 neutrals in 5 different positions), presented in a random sequence. The testing session consisted of 6 blocks, with breaks in between, and lasted approximately 35 minutes.

### Procedure

Prior to the experiment the volunteers gave their consent and filled out two questionnaires, the first being the ‘Edinburgh Handedness Questionnaire’ [43], and the second being a ‘Demographics and Health Questionnaire’. The Demographics and Health Questionnaire was primarily used to ensure the participants had no history of any psychological/psychiatric conditions (e.g., depression, anxiety) because these conditions may cause impaired expression recognition [44]. After filling the questionnaires, the participant’s visual acuity was measured using a Bailey-Lovie logMAR chart [45] at 3 meters, to ensure all participants had normal or corrected-to-normal vision (average binocular logMAR acuity = -0.04, standard deviation = 0.05).

Participants were seated in a dim room and shown a PowerPoint presentation to demonstrate the faces which would be used in the experiment and how each face looks when expressing the 6 different emotions and neutral. This was then followed by a training phase where faces were presented centrally on the screen and the participant decided whether the facial expression was happy, sad, fearful, disgusted, surprised, angry or neutral. In order to move on to the main experiments the participant needed to first discriminate facial emotions at least 70% on average (average proportion correct = 0.78, standard deviation = 0.05).

After the training, participants were asked to perform the two experiments (discrimination and detection, order counterbalanced). In each of the experiments a fixation cross was presented for 500ms. This was followed by face stimulus for 140ms, then a mask for 200ms. We used a relatively short presentation time for the face so that a saccadic eye movement cannot occur [46].

### Analysis

For each task, we used a modified form of the signal detection sensitivity measure d’. We used this as it provides a method to quantify sensitivity independently of response bias. For analysis of the recognition task we calculated a modified form of the *d’* sensitivity measure to determine participants’ sensitivity in discriminating a given expression from the remaining expressions, per eccentricity (see [6, 47]). A 5 x 7 repeated measures ANOVA with eccentricity and emotion as within-subject factors was subsequently performed. For analysis of the emotion detection task, we again used a modified form of the *d’* sensitivity measure as an objective method to determine participants’ sensitivity in discriminating a given expressive face from neutral. Hence in the detection task, the ANOVA analysis was based on the six basic expressions (excluding neutral), and thus a 5 X 6 repeated measures ANOVA was computed. Furthermore, where the two-way interaction was significant, we ran further one-way ANOVAs to identify at which eccentricities there was an effect of the Emotion factor. Where there was a significant effect of Emotion, we then computed follow-up paired sample t-tests to determine the significant differences between expression categories (21 possible comparisons). Control for multiple comparisons was achieved by use of the Bonferroni method (corrected *p* = 0.05 / 21).

In addition, in order to explicitly compare performance across the two tasks, we also conducted a three-way ANOVA with the factors: Task (2), Emotion (6), Eccentricity (5), where neutral faces were excluded from the Recognition task data for comparability with the detection task data. This allowed statistical analysis of whether task changes performance.

## Results

### 7AFC categorization task

**Fig 1:**
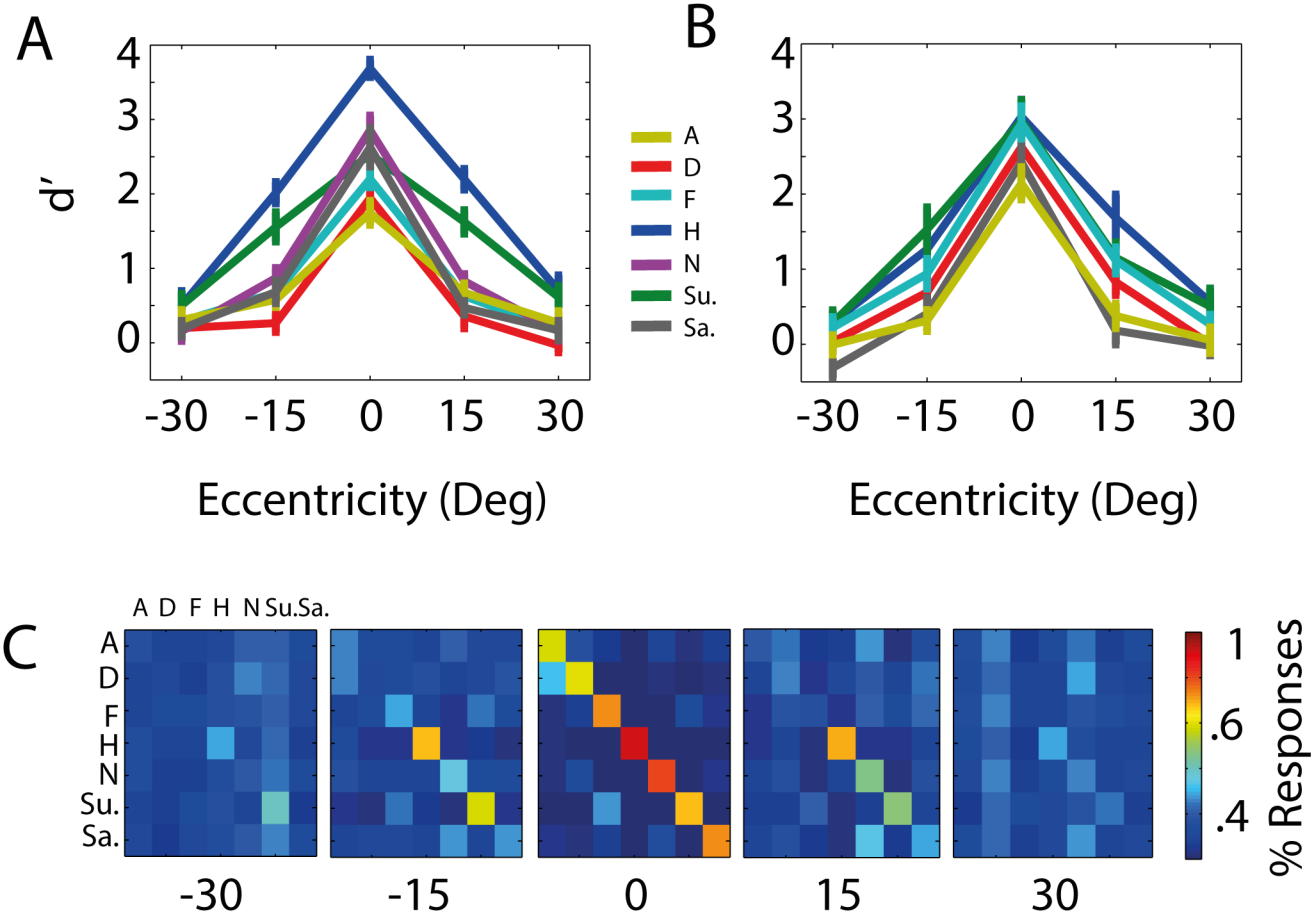
Performance in the expression recognition and detection tasks. (A) Expression Recognition: Average d’ scores as a function of eccentricity of stimulus presentation, for each basic facial expression category plus neutral (A = Anger; D = Disgust; F = Fear; H = Happy; N = Neutral; Su. = Surprise; Sa. = Sad). Error bars represent standard error of the mean. (B) Expression Detection: Average d’ scores as a function of eccentricity of stimulus presentation, for each basic facial expression category (A = Anger; D = Disgust; F = Fear; H = Happy; Su. = Surprise; Sa. = Sad). Error bars represent standard error of the mean. (C) Full confusion matrices underlying performance at each eccentricity for the Emotion Recognition Task (rows = expression presented; columns = response chosen).

Fig 1A shows the average d’ scores achieved in the recognition task as a function of each facial expression category and each eccentricity. A two by two (Emotion X Eccentricity) repeated measures ANOVA revealed main effects of both *Eccentricity*: *F* (4, 52) = 161.05, *p* < .001,${\text{partial}\;\eta }_{p}^{2}\;=\;0.93$, and *Emotion*, *F* (6, 78) = 32.32, *p* < .001, ${\text{partial}\;\eta }_{p}^{2}\;=\;0.71$, plus a significant interaction, *F* (24, 312) = 5.85, *p* < .001, ${\text{partial}\;\eta }_{p}^{2}\;=\;0.31$. In order to decompose the interaction, we computed separate one way ANOVAs for the effect of Emotion independently at each eccentricity. The effect of Emotion was significant at central fixation, 15 degrees to both sides of space, and 30 degrees to the right (all *p’s* < .003).

We then computed follow-up paired-sample t-tests comparing each expression category, independently for each eccentricity where the one-way ANOVA was significant, using the Bonferroni method to control for multiple comparisons (see Methods, corrected *p* = 0.05/21) and revealed that: at central fixation, happy faces (Mean = 3.69) were recognized significantly better than all other expressions (see Table 1). Neutral faces (Mean = 2.86) were recognized significantly better than angry, disgusted and fearful faces (Means = 1.75, 1.95, 2.20, respectively; see Table 1). Thus at central fixation, happy faces are clearly the best recognized expression.

**Table 1:** P values and effect sizes (in parentheses) from paired-sample t-tests between each pair of expressions at central fixation, for the recognition task.

Expression							
Expression	Angry	Disgusted	Fearful	Happy	Neutral	Surprised	Sad
Angry		.35(.26)	.014(.76)	<.001(3.14)^c^	<.001(1.45)^c^	.004 (.92)	.007(.86)^c^
Disgusted			.097(.48)	<.001(2.40)^c^	<.001(1.34)^c^	.026 (.67)	.069(.53)
Fearful				<.001(2.44)^c^	<.002(1.01)^c^	.151(0.41)	.134(.43)
Happy					<.001(1.18)^r^	<.001(2.04)^r^	.001(1.09)^r^
Neutral						.156 (.40)	.378 (.24)
Surprised							.787 (.07)

Note.

^c^ = column greater than row sig with Bonferroni correction;

^r^ = row greater than column sig with Bonferroni correction.

At 15 degrees to the left happy faces (Mean = 2.02) were significantly better recognized than all other expressions except surprised faces (Mean = 1.56), and surprised faces in turn were now recognized significantly better than angry, disgusted and fearful faces (Means = 0.58, 0.27, 0.67 respectively; see Table 2). In addition, neutral (Mean = 0.87), faces were recognized significantly better than disgusted faces (Table 2). At 15 degrees to the right, happy faces (Mean = 2.20) were again recognized significantly better than all expressions except surprised (Mean = 1.62), whilst surprised faces were recognized significantly better than all expressions except happy (see Table 3). Thus at 15 degrees into peripheral vision, happy faces are still the best recognized expression, but surprised faces emerge as being better recognized than many other expression categories. Finally, at 30 degrees to the right, surprised faces were recognized significantly better than disgusted faces (*t* (13) = 4.53, *p* = .0006; *d* = 1.21; Mean Surprised = 0.61, Mean Disgusted = -.03) while a strong trend was present for happy faces to be better recognized than disgusted faces which did not survive correction (*t* (13) = 3.64, *p* = .003; *d* = .97; Mean Happy = 0.71, Mean Disgusted = -.03).

**Table 2:** P values (effect sizes in parentheses) from paired-sample t-tests between each pair of expressions at -15 degrees eccentricity, for the recognition task.

Expression							
Expression	Angry	Disgusted	Fearful	Happy	Neutral	Surprised	Sad
Angry		.045(.59)	.57(.16)	<.001(2.39)^c^	.074 (.52)	<.001(1.47)^c^	.58(.15)
Disgusted			.009(.81)	<.001(2.89)^c^	.002 (1.06)^c^	<.001(1.43)^c^	.024(.68)
Fearful				<.001(2.41)^c^	.247(.32)	.001 (1.07)^c^	.95 (.02)
Happy					<.001(1.66)^r^	.029(.65)	<.001(1.97)^r^
Neutral						.005(.91)	.36 (.25)
Surprised							.003 (.96)

Note.

^c^ = column greater than row sig with Bonferroni correction;

^r^ = row greater than column sig with Bonferroni correction

**Table 3:** P values (effect sizes in parentheses) from paired-sample t-tests between each pair of expressions at 15 degrees eccentricity, for the recognition task.

Expression							
Expression	Angry	Disgusted	Fearful	Happy	Neutral	Surprised	Sad
Angry		.053(.57)	.82 (.06)	<.001(2.30)^c^	.19 (.37)	.001 (1.09)^c^	.18 (.38)
Disgusted			.16 (.39)	<.001(2.67)^c^	.014 (.76)	<.001(1.78)^c^	.56 (.16)
Fearful				<.001(2.39)^c^	.341(.26)	<.001(1.53)^c^	.37(.25)
Happy					<.001(2.75)^r^	.008 (.83)	<.001(2.57)^r^
Neutral						<.001(1.16)^c^	.006 (.88)
Surprised							<.001(1.64)^r^

Note.

^c^ = column greater than row sig with Bonferroni correction;

^r^ = row greater than column sig with Bonferroni correction

Thus, in summary, happy faces are well recognized both at central fixation and out to 15 degrees in both sides of space. However surprised faces are better recognized relative to other expressions in peripheral but not central vision. Fig 1C shows the full confusion matrices underlying performance at each eccentricity.

### 2AFC emotion detection task

Fig 1B shows the mean *d’* scores for each facial expression category as a function of each eccentricity in the emotion detection task. A two by two (Emotion X Eccentricity) repeated measures ANOVA on *d’* scores for each emotion (excluding neutral), revealed significant main effects of both factors: Eccentricity—*F* (4, 52) = 76.82, *p* < .001, ${\text{partial}\;\eta }_{p}^{2}\;=\;0.86$, Emotion—*F* (5, 65) = 21.23, *p* < .001, ${\text{partial}\;\eta }_{p}^{2}\;=\;0.62$, and a non-significant interaction, *F* (20, 260) *=* 1.48, *p* = .09, ${\text{partial}\;\eta }_{p}^{2}\;=\;0.10$. We conducted follow-up t-tests to investigate the main effect of Eccentricity further (corrected with Bonferroni method): these tests revealed that performance was significantly improved from 30 to 15 degrees and from 15 degrees to central fixation, independently on each side of space (all *p’s* < .002; all *d’s* > 0.98). Thus as expected performance improved from peripheral to central visual presentation. To further investigate the main effect of emotion, we conducted follow-up paired-sample t-tests (corrected with Bonferroni method, as above), comparing each pair of emotion categories averaged across eccentricities. These revealed that happy (Mean = 1.37) and surprised (Mean = 1.29) faces were both significantly detected better than angry, disgusted, and sad faces (Means = 0.54, 0.84, 0.56) respectively; see Table 4 for p values and effect size estimates). Fearful faces (Mean = 1.1), however, were also detected significantly better than angry, disgusted and sad faces. Finally disgusted faces were detected significantly better than angry faces. Thus happy, surprised and fearful faces were the best detected facial expressions.

**Table 4:** P values (effect sizes in parentheses) from paired-sample t-tests between each pair of expressions collapsed across eccentricities, in the detection task.

Expression					
Expression	Disgusted	Fearful	Happy	Surprised	Sad
Angry	.002 (1.02)^c^	<.001 (1.79)^c^	<.001 (1.42)^c^	<.001 (1.83)^c^	.72 (.097)
Disgusted		.001 (1.10)^c^	.001 (1.13)^c^	<.001 (1.43)^c^	.025 (.68)
Fearful			.015 (0.75)	.067 (0.53)	<.001 (1.23)^r^
Happy				.47 (0.20)	<.001 (1.41)^r^
Surprised					<.001 (1.56)^r^

Note.

^c^ = column greater than row sig with Bonferroni correction;

^r^ = row greater than column sig with Bonferroni correction

### Statistical comparison across tasks

In order to test for statistical differences on expression performance across the two tasks, we conducted a three-factor ANOVA with the factors Task (Recognition or Detection), Emotion (happy, surprised, disgusted, fearful, angry or sad faces) and Eccentricity (as above). Note this analysis was based on the six basic expressions only (i.e. excluding neutral, see Methods) and hence uses different data than the analyses reported above for the Recognition Task. The analysis revealed highly significant main effects of both Eccentricity—*F* (4, 52) = 136.56, *p* < .001, ${\text{partial}\;\eta }_{p}^{2}\;=\;0.91$, and Emotion—*F* (5, 65) = 44.1, *p* < .001, ${\text{partial}\;\eta }_{p}^{2}\;=\;0.77$, but there was no main effect of Task—*F* (1, 13) = .74, *p* = .404, ${\text{partial}\;\eta }_{p}^{2}\;=\;0.05$. The three way interaction between all factors was highly significant, *F* (20, 260) = 2.51, *p* = .001, ${\text{partial}\;\eta }_{p}^{2}\;=\;0.16$, and each two-way interaction also reached significance (all *p’s* < .035). To understand the three-way interaction, we conducted separate 2X2 ANOVAS (Eccentricity X Emotion) for each task.

As in the previous analyses (see independent analyses reported above), the three way interaction arose because of a highly significant Eccentricity X Emotion interaction in the Recognition Task, *F* (20, 260) = 6.19, *p* < .001, ${\text{partial}\;\eta }_{p}^{2}\;=\;0.32$, but no significant interaction on the Detection Task, *F* (20, 260) = 1.48, *p* = .09, ${\text{partial}\;\eta }_{p}^{2}\;=\;0.102$. Thus the pattern of which expressions can be well discriminated changes as a function of eccentricity only in the Recognition but not the Detection task.

In addition, the two-way interaction between Emotion and Task was highly significant, *F* (5, 65) = 3.24, *p* < .001, ${\text{partial}\;\eta }_{p}^{2}\;=\;0.41$. Although care must be exercised in interpretation of this effect in the presence of the higher order 3 way interaction, we explored the effect further by running paired-sample t-tests between each expression across tasks. Correction for multiple comparisons was achieved by using the Bonferroni method (corrected *p* = 0.05/6). Performance on fearful faces significantly improved in the detection Vs recognition task (*t* (13) = 4.51, *p* < .001, *d* = 1.20; Mean recognition = .79, Mean detection = 1.1) whereas performance on happy faces significantly decreased (*t* (13) = -3.70, *p* = .003, *d* = .99; Mean Recognition = 1.82, Mean Detection = 1.37). There was also a trend for better detection than recognition performance for disgusted faces which did not survive correction (*t*(13) = 2.67, *p* = .020, *d* = .71; Mean Recognition = .55, Mean Detection = .84). Thus task constraints influence how well specific facial expression signals can be perceived.

Finally as the two-way interaction between Task and Eccentricity was significant, we explored this effect further by running paired sample t-tests between each eccentricity within each task. Both tasks revealed a similar effect of eccentricity: performance significantly improved from 30 degrees to 15 degrees to central presentation, on both sides of space (all *p’s* < .003; all *d’s* > 0.98). We also examined the effect of task for each eccentricity but no effects were significant after correction for multiple comparisons (all *p’s* > = .023).

## Discussion

In the recognition task, happiness and surprise were generally the best recognized expressions in peripheral vision. In the detection task, we found that happiness and surprise again were well detected expressive faces. However, fearful faces were also well detected, and in fact showed significantly better detection than recognition performance. Thus our results reveal that certain basic expression categories are both well recognized and detected in peripheral vision (e.g. happiness, surprise) whereas others perform poorly in both tasks (e.g. anger, sadness), where still yet others show a different pattern in detection vs recognition tasks (e.g. fear). Thus our results show that task constraints shape expression perception in peripheral vision. Further, our results demonstrate that the pattern of which expressions can be discriminated interacts with eccentricity only in the recognition and not the detection task. This implies that expression differences in the recognition task are more dependent upon the higher spatial frequency content of the stimulus.

### Task changes how well specific facial expressions are perceived in peripheral vision

In the present study, we found happiness and surprise to be the best recognized expressions at further eccentricities. This fits with our previous findings of better recognition at greater viewing distances [6, 32]. The advantage for happy faces in peripheral vision agrees with the earlier work of [33–34]and extends their findings across the full set of basic expressions and much further into the visual periphery. Either presenting a face further in the periphery or increasing viewing distance degrade the object-based HSFs, hence the task must subsequently be performed with a greater reliance on the original object-based LSFs [17]. It is important to note that we found relatively poor *recognition* of fear in both the present work and in our previous work [6], see also [32]. Thus it does not seem that *recognition* of fear is at all special within the far periphery or at far viewing distances despite claims of the importance of its processing from object-based LSF information [22–23, 24]. However, in the current study we report that, in contrast, fear is a well detected emotion even in the visual periphery, and crucially that it is detected significantly better than it is recognized. This suggests that task constraints influence how well fear can be perceived, see also [31].

One crucial difference between the two tasks is that in detection, fearful faces do not need to be discriminated from surprised faces. There is a high confusion between these two emotions in recognition tasks, even in foveal vision (Fig 1C). Thus these considerations lead to the question of what is the primary task the brain engages in when confronted with an expressive face in the environment: does detection come first, before recognition? In [38] the authors proposed such an account for emotion perception and show how detection and recognition dissociate for a limited subset of the basic expressions. If such an account is true, then fearful face signals will readily be classed as in the important *emotion* category for subsequent analysis. But at what point might they need to be differentiated from surprised faces? The expression of both fear and surprise lead to a wider opening of the eyes (greater exposure of the sclera), and this in turn has been shown (in the case of fear) to benefit both the expresser and the observer in detecting important environmental events, especially in the near periphery (~9 deg) of the visual field [48], see also [49]. In addition, the underlying dynamic visual signals generated from facial muscle movements that comprise each expression are not discriminable early on during the signaling time-course, suggesting that fear and surprise are reducible to one basic expression category at early processing stages [50]. Surprise, furthermore, is also a somewhat unusual basic emotion category as it is typically only signaled for short intervals before evolving into a different expression (e.g. happily surprised, fearfully surprised and so on; see [51]) and it is not initially biased towards negative valence [52]. Thus fear and surprise will not necessarily have to be distinguished within an early time window, as both can be put into the same category of potentially conveying an immediate threat from an outside source. If this were the case, then one would predict that time-sensitive neuroimaging methods could be used to reveal similar representations for fear and surprise at early time periods, which only later diverge. Similar arguments can be made for why disgusted faces might show a different pattern of performance in the detection vs recognition task, as in recognition they are often confused with angry faces (see Fig 1C) but in detection this source of confusion is removed (see also [50]). Indeed in the present work, we found a strong trend for disgusted faces to be detected significantly better than they are recognized, although this did not survive multiple comparison correction.

Interestingly, our results also demonstrated the converse pattern for happy faces: i.e. better recognition than detection performance. It is important to note, however, that happy faces are still one of the best detected expressions (alongside surprised and fearful faces), and showed a strong trend for better detection than fearful faces (*p* = .015; see Table 4). Why should happy faces be more poorly detected than recognized? We speculate that this might be due to the confusability of happy faces with neutral faces, which is enhanced, in the detection task relative to the recognition task (as neutral faces comprise the only comparison category in the detection task). In our own recognition data, happy faces are confused with neutral faces in peripheral vision: at 30 degrees happy faces are misclassified as neutral at a rate of 14%, and neutral faces as happy at 9% whereas at 15 degrees these rates are subjectively lower, 8% and 4%, respectively. Similar confusions have been reported by [32]. These authors examined recognition of expression at different resolutions using the same stimulus set as in the present experiment, and reported that happy faces were confused with neutral faces at low resolutions (8% misclassification of neutral faces as happy and 9% misclassification of happy faces as neutral, at the lowest resolution reported). Hence there is precedent to suggest that neutral faces can be confused with happy faces, specifically when either spatial resolution is low in central vision or stimuli are presented in peripheral vision, and hence where in both cases only relatively lower stimulus-based LSFs are available. This might lead one to expect a significant interaction between Emotion and Eccentricity also on the detection task: although we did not report this interaction as being significant, there was a trend evident in our data (*p* = .09). As such the key point we want to stress is not that the effect of emotion on detection tasks is not affected by eccentricity but rather that it is *less* affected than on recognition tasks (see next section).

Our present findings provide strong converging evidence with the work of [31], in demonstrating that task constraints influence how well certain expressions can be perceived (fear & happiness). In [31] using the Bubbles classification image methodology, the authors showed that different visual information from the face was used for different expressive categorizations (e.g. Fear Vs Neutral, Vs 7AFC expression discrimination). In the present work we show that two important hypothesized stages of expression processing (i.e. emotion detection Vs recognition—see also e.g. [38]) lead to significant differences in how well certain expressions can be differentiated (i.e. fearful & happy faces). In particular [31] showed that a Fear Vs Neutral discrimination makes use of LSF stimulus content, whereas recognizing fear in the context of all basic expressions plus neutral (i.e. 7AFC) does not make the same use of LSF stimulus content. This provides a potential explanation of why fear is much better detected than recognized in the periphery, as stimulus-based LSFs are what remains available for analysis in peripheral vision.

### Recognition performance is more dependent upon eccentricity

We reported a highly significant three-way interaction between Eccentricity, Task and Emotion, which resulted from a significant interaction between Eccentricity and Emotion only in the recognition task, but not the detection task. This again demonstrates that task shapes the perception of facial expressions in peripheral vision. It further suggests that recognition of expression is more dependent upon the stimulus-based HSFs, which are gradually removed as a stimulus moves further into peripheral vision. This provides novel evidence that recognition and detection rely on partially separate underlying mechanisms, and complements recent work showing that the two tasks are differentially sensitive to configural Vs featural information from the face [38]. We note that there is evidence for the independence of face detection Vs identification [36], and also object detection Vs categorization [35, 53]; hence the present results together with those of [38] suggest that expression detection Vs recognition may also fit the same general pattern. In particular, if detection comes first, as suggested in [38], and relies to a greater extent on stimulus-based LSFs, as implied by our findings, that would permit operation of a fast detection mechanism (e.g. sub-cortical route, see [22–23]), whereas recognition, relying to a greater extent on stimulus-based HSFs would take more time to accomplish. These ideas could be tested in a future experiment that investigates speed of detection Vs recognition as a function of stimulus-based SF content.

Indeed it has been proposed that proceeding from coarse to fine SF information is a general strategy for visual information processing in the brain. In [54], for instance, it was shown that lower SF information drives FFA activity at short presentation times (75ms) while higher SF information leads to stronger responses with longer presentation times (150ms). Hence important brain regions for face perception show differential sensitivities to LSF Vs HSF information in a time dependent fashion.

### Implications for future studies

Our findings also lead us to consider the tasks and specific expressions that have been used in previous studies. At the behavioral level, in [16] the authors investigated detection of fear Vs neutral (and also disgust Vs neutral) at up to 40 degrees into the periphery. One explanation considered for why fear was well detected in peripheral vision in this study, was because it is more threat-related, and hence could make use of a rapid sub-cortical processing route based on magnocellular visual pathways ([16]; see also [22]). However, one important limitation of this study was that it only tested detection of two basic facial expression categories (fear Vs neutral and disgust Vs neutral). From our present findings, we reveal that fear is detected better than disgusted, angry and sad faces, and not significantly differently from surprised and happy faces (although there is a strong trend for happy faces to be better detected than fearful faces). Hence any implications of special processing of fear in the periphery, even in detection tasks, needs careful evaluation using additional emotion categories (e.g. at least surprised and happiness).

### Conclusion

In summary, in the present work we have shown that some expressions are both recognized and detected well in peripheral vision (i.e. happiness, surprise) whereas others are poorly recognized and detected (i.e. angry and sad) whereas yet others change profile as a function of task (e.g. fear). Our results show compellingly that task constraints shape the perception of expression in peripheral vision and provide novel evidence that detection and recognition rely on separate underlying mechanisms, with recognition being more dependent upon the stimulus-based HSFs. Finally, our work emphasizes the importance of considering the specific task and particular expression categories utilized when evaluating theoretical claims as to the importance of expression processing for particular basic emotion categories.

## Supporting information

S1 Dataset Data (d-prime scores) underlying the main statistical analyses for every participant in each task (recognition & detection) for each combination of emotion and eccentricity. (XLSX)
